# A Kinetic Finite Volume Discretization of the Multidimensional PIDE Model for Gene Regulatory Networks

**DOI:** 10.1007/s11538-023-01251-3

**Published:** 2024-01-22

**Authors:** Mihály A. Vághy, Irene Otero-Muras, Manuel Pájaro, Gábor Szederkényi

**Affiliations:** 1 Faculty of Information Technology and Bionics, Pázmány Péter Catholic University, Práter u. 50/a, Budapest, 1083 Hungary; 2 Institute for Integrative Systems Biology, Spanish Council for Scientific Research, Carrer del Catedràtic Agustín Escardino Benlloch, 46980 Valencia, Spain; 3 Department of Mathematics, Escola Superior de Enxeñaría Informática, University of Vigo, Campus Ourense, 32004 Ourense, Spain; 4 Systems and Control Laboratory, ELKH Institute for Computer Science and Control (SZTAKI), Kende u. 13-17, Budapest, 1111 Hungary

**Keywords:** Gene regulatory networks, Partial integro-differential equations, Compartmental systems, Kinetic systems, Numerical methods, Finite volume methods

## Abstract

In this paper, a finite volume discretization scheme for partial integro-differential equations (PIDEs) describing the temporal evolution of protein distribution in gene regulatory networks is proposed. It is shown that the obtained set of ODEs can be formally represented as a compartmental kinetic system with a strongly connected reaction graph. This allows the application of the theory of nonnegative and compartmental systems for the qualitative analysis of the approximating dynamics. In this framework, it is straightforward to show the existence, uniqueness and stability of equilibria. Moreover, the computation of the stationary probability distribution can be traced back to the solution of linear equations. The discretization scheme is presented for one and multiple dimensional models separately. Illustrative computational examples show the precision of the approach, and good agreement with previous results in the literature.

## Introduction

Gene expression is a fundamental biological process of actually realizing DNA information in the form of proteins in living organisms. Therefore, the (quantitative) modeling of gene expression has been in the focus of research during the last decades (Smolen et al. 2000; Ay and Arnosti 2011). Gene regulatory networks (GRNs) are complex mechanisms through which cells are able to react to internal and external signals in a controlled way (Peter and Davidson 2015). The set of techniques successfully applied for the modeling of GRNs is really wide (De Jong 2002; Karlebach and Shamir 2008; Barbuti et al. 2020). It was pointed out already in the 1970s that the stochastic nature of gene expression has to be taken into consideration during modeling (Berg 1978). Experimental results and model analysis clearly show that both translational and transcriptional bursting contribute to stochasticity in prokaryote and eukaryote gene expression (Kaern et al. 2005; Dar et al. 2015). It is also known that in many cases, stochasticity in gene expression is functionally advantageous, and it can even result in robust phenotypes (MacNeil and Walhout 2011). In Mackey and Tyran-Kamińska (2015), the dynamics of a bistable genetic switch was analyzed, and it was shown that the qualitative properties of the stationary distribution are not significantly influenced by stochastic terms describing bursting and degradation. A theoretically sound hybrid modelling and simulation framework was proposed and applied to study the transcriptional dynamics of an autoregulation loop and a toggle switch in Bokes et al. (2013). An interesting computation result is that there exist two qualitatively different scenarios called "fluctuating" and “simultaneous” in the behaviour of the toggle switch in the presence of gene expression noise.

The dynamical model studied in this paper is originated in Friedman et al. (2006), where a symbolic approach is proposed for describing the stationary distribution of protein concentration in living cells in the form of partial integro-differential equations (PIDEs). The model is based on the master equation, and considers protein production in random bursts (see, also Elgart et al. 2011; Dar 2012) extended by transcription autoregulation. Feasible stationary distributions for this PIDE model with a slightly modified transcription rate were derived and classified in Pájaro et al. (2015). The generalized Friedman (or multidimensional PIDE) model was later introduced in Pájaro et al. (2017) which describes the operation of a genetic circuit of *n* genes expressed into *n* different protein types. Since finding symbolic solutions for the stationary distributions of the generalized Friedman model is not straightforward due to its generality, Pájaro et al. (2017) proposed a numerical procedure for the computation. The approach is based on a semilagrangian method for the discretization of the PIDE, and the computational results show that it is suitable to describe the behaviour of a wide class of GRNs with several different regulatory interactions and protein degradation rates. The generalized Friedman model and the subsequently developed simulation framework SELANSI (Pájaro et al. 2017) has since been widely used for design (Sequeiros et al. 2022), identification (Sequeiros et al. 2022) and control (Bokes and Singh 2019; Fernández et al. 2022) of GRNs. In Pájaro et al. (2019) a truncated version of the master equation corresponding to a special version of the one-dimensional Friedman model was proposed. As we will show later, this can be formally seen as a semi-discretization of the PIDE and can be generalized to both variable degradation rates and multidimensional GRNs.

Compartmental models are used to describe the transmission of modeled entities (material, particles, vehicles, people, information, etc.) between physical or abstract storage units, called compartments (Haddad et al. 2010). Accordingly, the application field of compartmental systems is really wide including (bio)chemistry, pharmacokinetics, ecological, epidemiological and transportation modeling (Godfrey 1983). Mathematically, the descriptive power of compartmental models is quite good as they are capable of representing numerous complex dynamical phenomena (Rap 1986; Brown 1980). Additionally, one of their main benefits is the associated directed graph structure (called the compartmental graph) representing possible transitions, and the related strong results on qualitative dynamical properties (Jacquez and Simon 1993; Cobelli and Romanin-Jacur 1976). Naturally, there is a vast literature on the dynamics of linear compartmental systems and the algebraic properties of the corresponding compartmental matrices (see, e.g. Anderson 2013; Berman and Plemmons 1994; Meshkat et al. 2015; Bortner and Meshkat 2022). Another important related family of models is the class of chemical reaction networks (CRNs) or kinetic systems which includes dynamical models that can be formally represented by a set of transformations (reactions) between abstract chemical complexes. The theory of CRNs is an intensively studied area, where deep results have been achieved on the properties of kinetic systems such as the structure and stability of equilibria (Feinberg 2019; Angeli 2009; Chellaboina et al. 2009). Linear kinetic systems with one-species complexes are trivially compartmental, where the compartments correspond to chemical species, and the reaction graph is identical to the compartmental graph (Érdi and Tóth 1989).

Many hyperbolic conservation laws are derived in an integral form, which, in the case of sufficiently smooth solutions and fluxes, can be rewritten in their usual differential form (LeVeque 2002). However, many practical problems involve discontinuous solutions, where shocks or singularities can develop quickly even from smooth initial data. We highlight that the (generalized) Friedman model can have this problem too, see Sect. 5 for a special single gene example where the symbolic solution is known and has a singularity in the origin. Thus, numerical methods derived from the differential form, such as finite differences, are expected to lose accuracy near discontinuities. This problem can for example be mitigated by an appropriate Finite Volume Method (FVM) based on the integral form of the PDE. Instead of computing possibly unreliable pointwise approximations we define grid cells and approximate the cell averages of the solution. This approach introduces a clear compartmental interpretation of semi-discretized PDEs and can naturally capture the underlying conservation law, too (Eymard and Gallouët 2000). As a consequence, in many applications the result of an FVM is guaranteed to be nonnegative and conservative, thus it is bounded; that is, FVM can be robust to the singularities of the solution.

Motivated by the above mentioned preliminary results, the aim of this paper is to propose an efficient novel computational approach based on compartmental discretization for the numerical solution of the multidimensional PIDE model introduced in Pájaro et al. (2017). It is shown that the dynamics of the approximating model can be written as a nonnegative linear time-invariant ODE. Therefore, the stationary distribution can be computed by solving a set of linear equations. The structure of the paper is the following. In the next section we introduce the PIDE model and the compartmental and kinetic systems. Next, in Sect. 3, we describe the kinetic finite volume discretization proposed to solve the PIDE model. A qualitative analysis of the proposed method is addressed in Sect. 4 and several illustrative numerical examples are shown in Sect. 5. Finally, in Sect. 6, we end up with a summary of the work.

## Notations and Background

In this section we give a brief introduction of multidimensional GRNs and (linear) compartmental and kinetic systems.

### Multidimensional Gene Regulatory Networks

The following short introduction is based on Friedman et al. (2006), Pájaro et al. (2017). We consider a gene regulatory network consisting of *n* genes $G=\{DNA_1,DNA_2,\dots ,DNA_n\}$ that express *n* proteins $X=\{X_1,X_2,\dots ,X_n\}$ via the corresponding messenger RNAs $M=\{mRNA_1,mRNA_2,\dots ,mRNA_n\}$. We follow the still relevant central dogma of molecular biology, which asserts that the gene instructions are transcribed into messenger RNAs, that are translated into proteins. The continuous number of mRNA molecules and proteins are denoted by ${\varvec{m}},{\varvec{x}}\in {\mathbb {R}}^n$, respectively.

The promoters corresponding to each gene are assumed to switch between active and inactive states, denoted by $DNA_{i,\text {on}}$ and $DNA_{i,\text {off}}$, respectively. The transition is controlled by the binding of proteins. Note that in general, the feedback mechanism may require the binding of multiple types of proteins besides the one expressed by the given gene. For the sake of generality, we assume that any protein can repress or activate any gene in the network. This mechanism is typically modelled by multivariate Hill functions. We define the matrix $H\in {\mathbb {Z}}^{n\times n}$, where $H_{ij}$ represents the Hill coefficient of the cross-regulation. If $H_{ij}$ is positive (respectively, negative), then $X_j$ inhibits (respectively, promotes) the expression of $X_i$.

The transcription of $DNA_i$ into $mRNA_i$ is assumed to be a first order process occurring with rate $k_m^i$ per unit time and with transcriptional leakage $\varepsilon _i\in (0,1)$. Then the transcription can be written as

$$\begin{aligned} R_T^i({\varvec{x}})=k_m^ic_i({\varvec{x}}), \end{aligned}$$

where $c_i:{\mathbb {R}}_+^n\rightarrow [\varepsilon _i,1]$ depend on the feedback regulation mechanism. In a single gene setting we could consider

$$\begin{aligned} c_1(x_1)=\frac{K^H+\varepsilon _1x^H}{K^H+x^H}, \end{aligned}$$

for some appropriate *K* and *H* constants; see Sect. 5 for more examples of $c_i$ Hill functions. We emphasize that the transcriptional leakage directly affects the range of these coefficient functions. Finally, the translation rate of protein $X_i$ is defined as

$$\begin{aligned} R_X^i(m_i)=k_x^im_i. \end{aligned}$$

The messenger RNA and protein degradation is assumed to take the form

$$\begin{aligned} G_m^i(m_i)=-\gamma _m^im_i\qquad G_X^i({\varvec{x}})=-\gamma _x^i({\varvec{x}})x_i, \end{aligned}$$

where $\gamma _m^i>0$ and $\gamma _x^i:{\mathbb {R}}_+^n\longrightarrow {\mathbb {R}}_+$. Following Pájaro et al. (2017) it is assumed that $\frac{\gamma _m^i}{\gamma _x^i({\varvec{x}})}\gg 1$ in order to ensure the validity of the subsequent model.

We use the standard exponential distribution to model protein bursting; that is, the conditional probability of the protein level jumping from $y_i>0$ to $x_i>y_i$ is

$$\begin{aligned} \omega _i(x_i-y_i)=\frac{1}{b_i}\exp \left[ -\frac{x_i-y_i}{b_i}\right] , \end{aligned}$$

where $$b_i=\frac{k_x^i}{\gamma _m^i}$$.

With the above assumptions the probability density function (PDF) of the protein level, $p(t,{\varvec{x}})$, can be modelled with the following PIDE:

1 $$\begin{aligned} \frac{\partial p(t,{\varvec{x}})}{\partial t}=\sum _{i=1}^n\frac{\partial }{\partial x_i}\big [\gamma _x^i({\varvec{x}}) x_ip(t,{\varvec{x}})]+\sum _{i=1}^nk_m^i\int _0^{x_i}\beta _i(x_i-y_i)c_i(\varvec{y_i}) p(t,\varvec{y_i})\textrm{d}y_i,\nonumber \\ \end{aligned}$$

where $\varvec{y_i}={\varvec{x}}+(y_i-x_i)e_i$ and the $\beta _i$ functions have the following form:

$$\begin{aligned} \beta _i(x)=\omega _i(x)-\delta (x). \end{aligned}$$

In Cañizo et al. (2019) the authors show the well-posedness of (1) in the generalized (mild) sense; that is, for $p_0\in {\mathcal {L}}^1({\mathbb {R}}^n)$ there exists a unique mild solution $p\in {\mathcal {C}}\left( {\mathbb {R}}_+;{\mathcal {L}}^1({\mathbb {R}}^n)\right)$ with the following properties:

(i) nonnegativity: if $p_0$ is nonnegative, then so is the solution *p*(*t*, .) for all $t\ge 0$,
(ii) mass conservation:
  $$\begin{aligned} \int _{{\mathbb {R}}_+^n}p(t,{\varvec{x}})\textrm{d}{\varvec{x}}=\int _{{\mathbb {R}}_+^n}p_0({\varvec{x}}) \textrm{d}{\varvec{x}},\qquad \forall t\ge 0. \end{aligned}$$

In fact, if the initial data is in the space ${\mathcal {C}}^{1,b}({\mathbb {R}}_+^n)$ of Hölder-continuous functions for some appropriate $b>0$ (e.g., in one dimension $b=b_1$), then there exists a unique classical solution $p\in {\mathcal {C}}^1\left( {\mathbb {R}}_+;{\mathcal {L}}^1({\mathbb {R}}_+^n)\right)$. Note, that in the probabilistic setting in applications we usually assume that $p_0$ is nonnegative and its integral is one.

### Compartmental and Kinetic Systems

We briefly introduce compartmental systems based on Jacquez and Simon (1993). Compartmental differential equations are often used to model physical phenomena governed by a conservation law such as conservation of mass. A compartment can represent a certain amount of a material that is kinetically homogeneous; that is, the entering material is instantly mixed with that of the compartment. As long as we can interpret the conservation law, a compartment can even describe abstract quantities, such as probabilities in our case. Despite this, we will usually refer to the amount of the modeled quantity in the compartment as the mass in the compartment, and to the conservation law as conservation of mass.

Let us consider a system with *m* compartments and let $q_i$ represent the mass of the *i*th compartment. In general, any compartment can be connected to any other compartment and to the environment in both directions. We denote with $F_{ij}$ the flow from the compartment $q_j$ to the compartment $q_i$, with $I_i$ the material inflow from the environment to compartment $q_i$ and with $F_{0i}$ the material outflow from compartment $q_i$ to the environment. Loop flows are not allowed, i.e. $i\ne j$ in $F_{ij}$. Then the time-evolution of the system is given by the following system of differential equations:

2 $$\begin{aligned} \dot{q}_i=\sum _{j\ne i}(-F_{ji}+F_{ij})+I_i-F_{0i}. \end{aligned}$$

We impose the following physical assumptions to the system:

1. for any $i,j,t\ge 0$, $i\ne j$ we have that $F_{ij}(q(t))\ge 0$, $I_i(t)\ge 0$ and $F_{0i}(q(t))\ge 0$,
2. for any $i,t\ge 0$ if $q_i(t)=0$, then $F_{0i} (q(t))=F_{ji} (q(t))=0$ for each *j*.

These properties ensure the invariance of the nonnegative orthant; that is, assuming a nonnegative initial condition, our solution is guaranteed to be nonnegative. In general, the above functions can depend on the mass of any compartment and possibly on *t* as well. Then it can be shown that if each $F_{ij}$ and $F_{0i}$ belong to the class ${\mathcal {C}}^k$, then we can rewrite (2) as

3 $$\begin{aligned} \dot{q}_i=-\left( f_{0i}+\sum _{j\ne i}f_{ji}\right) q_i+\sum _{j\ne i}f_{ij}q_j+I_i, \end{aligned}$$

where $F_{ij}=f_{ij}q_j$ and the fractional transfer coefficients $f_{ij}$ belong to the class ${\mathcal {C}}^{k-1}$, for $k\ge 1$. We can then naturally rewrite (3) in matrix form as

$$\begin{aligned} \dot{q}=fq+I, \end{aligned}$$

where $\{f\}_{ij}=f_{ij}$ for $i=0,1,\dots$ and $j=1,2,\dots$. If each fractional transfer coefficient $f_{ij}$ only depends on $q_j$, then the system is called a donor controlled system. If each coefficient is constant, then the system is called a linear donor controlled system.

Linear donor controlled systems can naturally be represented as chemical reaction networks, or kinetic systems. For a brief introduction, we refer to Angeli (2009). For each compartment with index *i*, $q_i$ represents the mass (or alternatively, the concentration) of the one-species complex $Q_i$, and for each transition from compartment *i* to *j*, we assign the reaction $Q_i\rightarrow Q_j$. Using this construction, we can not only rely on the comprehensive theory of compartmental models but on that of kinetic systems as well. While most qualitative properties of linear donor controlled systems we consider can be derived from both modeling approaches, a notable piece of additional information in chemical reaction network theory is the stability analysis using a logarithmic Lyapunov function, discussed in more detail in 4.2.

## Kinetic Finite Volume Discretization

In this section we formulate a finite volume discretization of (1), the result of which is a mass conservative kinetic system. We also note that since (1) is linear (that is, if *p* and *q* are solutions, then so is $p+q$), the result of the semi-discretization is anticipated to also be linear.

### One-Dimensional Case

Let us first consider the one-dimensional Friedman model describing the temporal evolution of protein distribution given by

4 $$\begin{aligned} \frac{\partial p(t,x)}{\partial t}=\frac{\partial }{\partial x}\left[ \gamma _x^1(x)xp(t,x)\right] +k_m^1\int _0^x\beta _1(x-y)c_1(y)p(t,y)\textrm{d}y, \end{aligned}$$

with initial condition $p(0,x)=p_0(x)$ that has integral one. The mass conservation of (4) is well-known but the subsequent informal investigation provides further insight that can be transferred to the design of the numerical scheme. Since $\omega _1$ is the probability density function of an exponential distribution its integral is one, and thus

$$\begin{aligned} \int _0^{\infty }\beta _1(x)\textrm{d}x=0. \end{aligned}$$

Integrating over ${\mathbb {R}}_+$ shows after a change of variables that

5 $$\begin{aligned} \begin{aligned}&\int _0^{\infty }\frac{\partial p(t,x)}{\partial t}\textrm{d}x=\frac{\partial }{\partial t}\int _0^{\infty }p(t,x)\textrm{d}x=\int _0^{\infty }\ \frac{\partial }{\partial x}\left[ \gamma _x^1(x)xp(t,x)\right] \textrm{d}x\\&\qquad +k_m^1\int _0^{\infty }\int _0^x\beta _1(x-y)c_1(y)p(t,y)\textrm{d}y\textrm{d}x=\underbrace{\lim _{x\rightarrow \infty } \gamma _x^1(x)xp(t,x)}_{=0}-\gamma _x^1\cdot 0\cdot p(t,0)\\&\qquad +k_m^1\int _0^{\infty }\int _y^{\infty }\beta _1(x-y)c_1(y)p(t,y)\textrm{d}x\textrm{d}y\\&\quad =k_m^1\int _0^{\infty }c_1(y)p(t,y)\int _y^{\infty }\beta _1(x-y)\textrm{d}x\textrm{d}y=0, \end{aligned} \end{aligned}$$

so that the equality

$$\begin{aligned} \int _0^{\infty }p(t,x)\textrm{d}x=\int _0^{\infty }p_0(x)\textrm{d}x=1 \end{aligned}$$

holds for any $t\ge 0$.

In a finite volume setting the coefficients are calculated as averages (that is, integrals) over appropriate subdomains. Hence, as an intuition we should note that the mass conservation property of the novel scheme should be the result of a calculation very similar to (5).

Our main goal is to perform a spatial discretization (with resolution *h*) to obtain an infinite dimensional dynamical system describing the temporal evolution of the functions ${p_i(t)}_{i\in {\mathbb {N}}}$ with the usual properties of a PDF; that is, we should have that:

1. $0\le p_i(t)$ for all $i\in {\mathbb {N}}$ and $t\ge 0$,
2. $$\sum _{i=1}^{\infty }hp_i(t)=1$$ for all $$t\ge 0$$.

In order to do so, consider the set of intervals

$$\begin{aligned} K_i=\left[ x_{i-\frac{1}{2}},x_{i+\frac{1}{2}}\right] =\left[ (i-1)h,ih\right] ,~i=1,2,\dots \end{aligned}$$

for some $h>0$ and introduce the set of variables $p_i(t)$, where

$$\begin{aligned} p_i(t)\approx \frac{1}{|K_i|}\int _{K_i}p(t,y)\textrm{d}y=\frac{1}{h}\int _{K_i}p(t,y)\textrm{d}y; \end{aligned}$$

that is, the value $p_i(t)$ is assumed to approximate the average in the cell $K_i$ and we set the initial values accordingly. Further introduce the cell averages of the functions $\gamma _x^1$ and $c_1$ given as

$$\begin{aligned} \gamma _i^1=\frac{1}{|K_i|}\int _{K_i}\gamma _x^1(y)\textrm{d}y,\qquad c_i^1=\frac{1}{|K_i|}\int _{K_i}c_1(y)\textrm{d}y. \end{aligned}$$

Let $x_i$ be the midpoint of $K_i$ for $i=1,2,\dots$ and define

$$\begin{aligned} \begin{aligned} b_{i,i}^1&=\frac{1}{h/2}\int _{[(i-1)h,(i-1/2)h]}\beta _1(x_i-y)\textrm{d}y=\frac{1}{h/2} \int _{[x_i-h/2,x_i]}\beta _1(x_i-y)\textrm{d}y,\\ b_{i,j}^1&=\frac{1}{|K_j|}\int _{K_j}\beta _1(x_i-y)\textrm{d}y,\quad j=1,2,\dots ,i-1. \end{aligned} \end{aligned}$$

As the $b_{i,j}^1$ coefficients come from a discretization of an exponential function, the series is geometric (apart from $b_{i,i}^1$ that takes the Dirac delta into account), see Berg (1978), Bokes et al. (2011), McAdams and Arkin (1997). As the derivative on the right-hand side of (4) describes protein degradation (that is, a vector field pointing towards the origin) we will approximate it with a difference quotient of the form

$$\begin{aligned} \frac{\partial }{\partial x}\left[ \gamma _x^1(x)xp(t,x)\right] \Bigg |_{K_i}\approx \frac{1}{h}\left( \gamma _{i+1} ^1x_{i+\frac{1}{2}}p_{i+1}(t)-\gamma _i^1x_{i-\frac{1}{2}}p_i(t)\right) . \end{aligned}$$

Then approximating the integral in (4) with a sum yields the system

6 $$\begin{aligned} \begin{aligned} \dot{p}_i(t)&=\frac{1}{h}\left( \gamma _{i+1}^1x_{i+\frac{1}{2}}p_{i+1}(t) -\gamma _i^1x_{i-\frac{1}{2}}p_i(t)\right) +k_m^1\sum _{j=1}^ih_{i,j}^1b_{i,j}^1c_j^1p_j(t);\\ p_i(0)&=\frac{1}{|K_i|}\int _{K_i}p_0(y)\textrm{d}y, \end{aligned} \end{aligned}$$

where

$$\begin{aligned} h_{i,j}^1={\left\{ \begin{array}{ll}h/2,\quad &{}i=j,\\ h,\quad &{}i\ne j.\end{array}\right. } \end{aligned}$$

Observe, that the resulting infinite dimensional system (6) is clearly a linear donor controlled compartmental system of the form

$$\begin{aligned} \dot{p}(t)=\Gamma p(t), \end{aligned}$$

where the infinite matrix defined element-wise as

$$\begin{aligned} \Gamma _{ij}={\left\{ \begin{array}{ll} k_m^1h_{i,j}^1b_{i,j}^1c_j^1,\quad &{}j<i,\\ -\frac{1}{h}\gamma _i^1x_{i-\frac{1}{2}}+k_m^1h_{i,i}^1b_{i,i}^1c_i^1,\quad &{}j=i,\\ \frac{1}{h}\gamma _{i+1}^1x_{i+\frac{1}{2}},\quad &{}j=i+1,\\ 0,\quad &{}j>i+1 \end{array}\right. } \end{aligned}$$

is an infinite Kirchhoff matrix; that is, it is a Metzler matrix with zero column-sums. While the superdiagonal elements of $\Gamma$ can be arbitrarily large, its sign structure guarantees the well-posedness, in the $\ell ^1$ space of absolutely summable sequences, of the underlying continuous-time infinite Markov chain of (6), see Sects. 4 and 6 of Reuter (1957). This further implies the well-posedness of (6), and thus relying on the fact that $\beta _1$ integrates to zero we have that

$$\begin{aligned}&\sum _{i=1}^{\infty }\dot{p}_i(t)=\sum _{i=1}^{\infty }\frac{1}{h}\left( \gamma _{i+1}^1x_{i+\frac{1}{2}}p_{i+1}(t) -\gamma _i^1x_{i-\frac{1}{2}}p_i(t)\right) +k_m^1\sum _{i=1}^{\infty }\sum _{j=1}^ih_{i,j}^1b_{i,j}^1c_j^1p_j(t)\\&\quad =\lim _{l\rightarrow \infty }\frac{1}{h}\gamma _{l+1}^1x_{l+\frac{1}{2}}p_{l+1}(t)-\frac{1}{h}\gamma _1^1\cdot 0\cdot p_1(t)+k_m^1\sum _{j=1}^{\infty }\sum _{i=j}^{\infty }h_{i,j}^1b_{i,j}^1c_j^1p_j(t)\\&\quad =k_m^1\sum _{j=1}^{\infty }c_j^1p_j(t)\sum _{i=j}^{\infty }h_{i,j}^1b_{i,j}^1\\&\quad =k_m^1\sum _{j=1}^{\infty }c_j^1p_j(t)\left( \int _{[x_j-h/2,x_j]}\beta _1(x_j-y)\textrm{d}y +\sum _{i=j+1}^{\infty }\int _{K_j}\beta _1(x_i-y)\textrm{d}y\right) \\&\quad =k_m^1\sum _{j=1}^{\infty }c_j^1p_j(t)\left( \int _0^{h/2}\beta _1(y)\textrm{d}y+\sum _{i=j+1} ^{\infty }\int _{[(i-j-1/2)h,(i-j+1/2)h]}\beta _1(y)\textrm{d}y\right) \\&\quad =k_m^1\sum _{j=1}^{\infty }c_j^1p_j(t)\int _0^{\infty }\beta _1(y)\textrm{d}y=0, \end{aligned}$$

so that the equality

$$\begin{aligned} \sum _{i=1}^{\infty }hp_i(t)=\sum _{i=1}^{\infty }hp_i(0)=1 \end{aligned}$$

holds for any $t\ge 0$. The above facts combined also show that $p_i(t)\le \frac{1}{h}$ for any $t\ge 0$.

#### Remark 1

The above calculation shows that the conservativity of the scheme only requires that the $b_{i,j}^1$ coefficients are averages of the $\beta _1$ function; that is, the $\gamma _i^1$ and $c_i^1$ coefficients could be just point values of the underlying coefficient functions on the mesh points. Nevertheless, taking the averages might be more precise if those functions change rapidly.

### Multidimensional Case

Let us consider the multidimensional model (1) with $n>1$. Define the positive step sizes $h_1,h_2,\dots ,h_n$ and setswhere $\alpha \in {\mathbb {N}}^n$ is a multi-index. Let us note that each cell has the same size and define $h=|K_{\alpha }|=\prod _{i=1}^nh_i$. Similarly to the one-dimensional case, for each cell $K_{\alpha }$ we introduce the function $p_{\alpha }(t)$ assumed to approximate the cell average as

$$\begin{aligned} p_{\alpha }(t)\approx \frac{1}{h}\int _{K_{\alpha }}p(t,{\varvec{y}})\textrm{d}{\varvec{y}}. \end{aligned}$$

For $i=1,2,\dots ,n$ we also compute the variables

$$\begin{aligned} \begin{aligned} \gamma _{\alpha }^i&=\frac{1}{h}\int _{K_{\alpha }}\gamma _x^i({\varvec{y}})\textrm{d}{\varvec{y}},\\ c_{\alpha }^i&=\frac{1}{h}\int _{K_{\alpha }}c_i({\varvec{y}})\textrm{d}{\varvec{y}}. \end{aligned} \end{aligned}$$

Let $x_{\alpha }=[x_{\alpha }^1~x_{\alpha }^2~\dots ~x_{\alpha }^n]^{\textrm{T}}$ be the midpoint (w.r.t. each dimension) of $K_{\alpha }$ and $x_{\alpha }^{i\pm \frac{1}{2}}=x_{\alpha }^i\pm \frac{h_i}{2}$; that is, the variables $x_{\alpha }^{i\pm \frac{1}{2}}$ correspond to the coordinates of the boundaries of $K_{\alpha }$. For $i=1,2,\dots ,n$ define

$$\begin{aligned} \begin{aligned} b_{\alpha ,\alpha _i}^i&=\frac{1}{h_i/2}\int _{[(i-1)h_i,(i-1/2)h_i]}\beta _i(x_{\alpha }^i-y)\textrm{d}y =\frac{1}{h_i/2}\int _{[x_{\alpha }^i-h_i/2,x_{\alpha }^i]}\beta _i(x_{\alpha }^i-y)\textrm{d}y,\\ b_{\alpha ,j}^i&=\frac{1}{h_i}\int _{[(j-1)h_i,jh_i]}\beta _i(x_{\alpha }^i-y)\textrm{d}y,\qquad j=1,2,\dots ,\alpha _i-1. \end{aligned} \end{aligned}$$

Similarly to the one-dimensional case the derivatives are approximated with difference quotients of the form

$$\begin{aligned} \frac{\partial }{\partial x_i}\left[ \gamma _x^i({\varvec{x}})x_ip(t,{\varvec{x}})\right] \Bigg |_{K_{\alpha }} \approx \frac{1}{h_i}\left( \gamma _{\alpha +e_i}^ix_{\alpha }^{i+\frac{1}{2}}p_{\alpha +e_i}(t) -\gamma _{\alpha }^ix_{\alpha }^{i-\frac{1}{2}}p_{\alpha }(t)\right) . \end{aligned}$$

Approximating the integrals in (1) with sums as before, yields the system

7 $$\begin{aligned} \begin{aligned} \dot{p}_{\alpha }(t)&=\sum _{i=1}^n\frac{1}{h_i}\left( \gamma _{\alpha +e_i}^ix_{\alpha } ^{i+\frac{1}{2}}p_{\alpha +e_i}(t)-\gamma _{\alpha }^ix_{\alpha }^{i-\frac{1}{2}}p_{\alpha }(t)\right) \\&+\sum _{i=1}^nk_m^i\sum _{j=1}^{\alpha _i}h_{\alpha ,j}^ib_{\alpha ,j}^ic_{\varvec{\alpha _{i,j}}} ^ip_{\varvec{\alpha _{i,j}}}(t);\\ p_{\alpha }(0)&=\frac{1}{h}\int _{K_{\alpha }}p_0({\varvec{y}})\textrm{d}{\varvec{y}}, \end{aligned} \end{aligned}$$

where $$\varvec{\alpha _{i,j}}=\alpha +(j-\alpha _i)e_i$$ and

$$\begin{aligned} h_{\alpha ,j}^i={\left\{ \begin{array}{ll}h_i/2,\qquad &{}j=\alpha _i,\\ h_i,\qquad &{}j\ne \alpha _i.\end{array}\right. } \end{aligned}$$

Again, the system is clearly kinetic and the mass conservation follows from a calculation very similar to the one-dimensional case:

$$\begin{aligned} \begin{aligned}&\sum _{\alpha }\dot{p}_{\alpha }(t)=\sum _{\alpha }\sum _{i=1}^n\frac{1}{h_i}\left( \gamma _{\alpha +e_i}^ix_{\alpha } ^{i+\frac{1}{2}}p_{\alpha +e_i}(t)-\gamma _{\alpha }^ix_{\alpha }^{i-\frac{1}{2}}p_{\alpha }(t)\right) \\&\qquad +\sum _{\alpha }\sum _{i=1}^nk_m^i\sum _{j=1}^{\alpha _i}h_{\alpha ,j}^ib_{\alpha ,j}^ic_{\varvec{\alpha _{i,j}}} ^ip_{\varvec{\alpha _{i,j}}}(t)\\&\quad =\sum _{\alpha }\sum _{i=1}^nk_m^i\sum _{j=\alpha _i}^{\infty }h_{\varvec{\alpha _{i,j}},j} ^ib_{\varvec{\alpha _{i,j}},j}^ic_{\alpha }^ip_{\alpha }(t)=\sum _{i=1}^nk_m^i\sum _{\alpha } c_{\alpha }^ip_{\alpha }(t)\underbrace{\sum _{j=\alpha _i}^{\infty }h_{\varvec{\alpha _{i,j}},j} ^ib_{\varvec{\alpha _{i,j}},j}^i}_{=0}=0. \end{aligned} \end{aligned}$$

This shows for any $t\ge 0$ that

$$\begin{aligned} \sum _{\alpha }hp_{\alpha }(t)=\sum _{\alpha }hp_{\alpha }(0)=1, \end{aligned}$$

further implying that $p_{\alpha }(t)\le \frac{1}{h}$ for each $\alpha$.

### Discretization on a Truncated Domain

In practical applications we may assume that there can only be a finite number of proteins of each kind. This consideration is naturally backed by the fact that the solution of (1) is integrable so that $\lim _{\Vert {{\varvec{x}}} \Vert _{{\mathbb {R}}^n}\rightarrow \infty }p(t,{\varvec{x}})=0$ for any $t\ge 0$. Thus, we discretize over the finite domain  for appropriately large $L_i>0$ values. According to these considerations we also assume that $\int _{\Omega }p_0({\varvec{x}})\textrm{d}{\varvec{x}}=1$.

We divide the $(0,L_i)$ intervals into $N_i$ equal subintervals and proceed to calculate the variables $p_{\alpha }(0)$ and the coefficients $\gamma _{\alpha }^i$ and $c_j^i$ as before. We similarly compute $b_{\alpha ,j}^i$ for $j=1,2,\dots ,\alpha _i-1$, but modify $b_{\alpha ,\alpha _i}^i$ to capture the fact that the number of *i*th kind of protein is maximalized in $L_i$.

Note, that the resulting system can still be given by (7) with the difference that the set of variables $\{p_{\alpha }\}$ is finite. While the bursts and degradations inherently define some “spatial” structure between the $p_{\alpha }$ variables (discussed in detail later), it might be more useful to think of the truncated semi-discretized model as a flattened *N*-dimensional system of the form

8 $$\begin{aligned} \dot{p}(t)=\tilde{\Gamma }^{(N)}p(t) \quad \text {with } N:=\prod _{i=1}^nN_i. \end{aligned}$$

## Qualitative Analysis

In this section we show that the result of the truncated kinetic finite volume discretization is not only a mass conservative nonnegative system but it has several advantageous qualitative properties.

### Structural Descriptions

While we could rely on the linearity of (8) to investigate its dynamical behaviour, the large number of variables and the complexity of the coefficient matrix $\tilde{\Gamma }^{(N)}$ renders this approach futile. Instead, let us focus on the inner structure of the system through its compartmental and CRN representations. These observations will immediately imply most qualitative properties of interest.

**Compartmental representation**

Consider the *N*-dimensional truncated system of the form (7). Based on the burst and degradation structure the system has a compartmental topology as follows:

- Each compartment $K_{\alpha }$ has an incoming edge from $K_{\alpha +e_i}$ due to protein degradation if $\alpha _i<N_i$ for $i=1,2,\dots ,n$.
- Each compartment $K_{\alpha }$ has an incoming edge from $K_{\varvec{\alpha _{i,j}}}$ for $i=1,2,\dots ,n$ and $j=1,2,\dots ,\alpha _i-1$ due to protein production in bursts.

Clearly, the compartmental topology is strongly connected, which property is essential for our further analysis. Based on this structure (and the flattening method) one can easily determine the elements of the matrix $\tilde{\Gamma }^{(N)}\in {\mathbb {R}}^{N\times N}$ of (8).

**Fig. 1:**
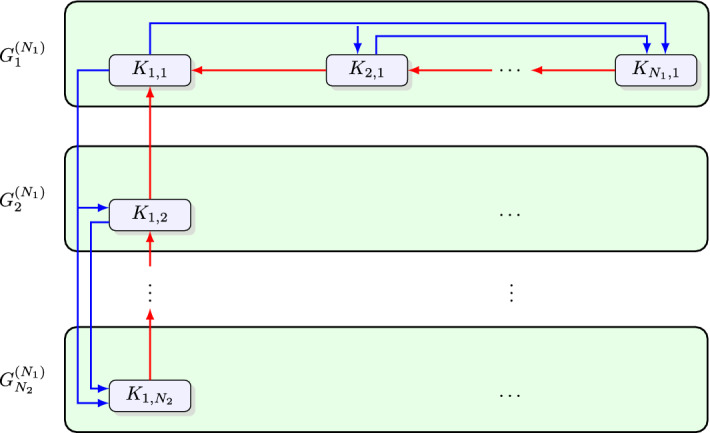
Compartmental topology of a two-dimensional model. Each subsystem is isomorphic to that of a one-dimensional model

To gain further insight into the compartmental topology, let us focus on some low-dimensional (in terms of the PIDE) examples. Figure 1 shows the structure of compartments for a two-dimensional PIDE. Degradations and bursts are denoted with red and blue arrows, respectively. Let $G^{(N_1,N_2)}$ denote the graph in Fig. 1; that is, a compartmental graph of appropriate size corresponding to (8). Notice, that the graph $G^{(N_1,N_2)}$ can be decomposed to the interconnected $G_1^{(N_1)},G_2^{(N_1)},\dots ,G_{N_2}^{(N_1)}$ graphs that are isomorphic to the compartmental graph of a one-dimensional model of size $N_1$. This shows that $G^{(N_1,N_2)}$ is isomorphic to the Cartesian product $G^{(N_1)}\times G^{(N_2)}$. In fact, the $G^{(N_1,N_2,\dots ,N_n)}$ compartmental graph of an *n*-dimensional model (8) is isomorphic to .

**CRN representation**

For each continuous variable $p_{\alpha }$ we introduce the species $P_{\alpha }$ and assign the complex $P_{\alpha }$ to the compartment $K_{\alpha }$. Then the complex composition matrix containing the stoichiometric coefficients of the complexes as its columns is the identity matrix $I\in {\mathbb {R}}^{N\times N}$, and the reaction structure is identical to the above compartmental topology; that is, the reaction graph is identical (isomorphic) to the compartmental graph and, in particular, is strongly connected. This readily shows that the deficiency of the reaction graph, as defined in CRN theory (Feinberg 2019), is zero as there are *N* distinct complexes, one linkage class and a spanning tree in the reaction graph of size $N-1$. Since the system is linear, the reaction vectors corresponding to the edges of the spanning tree span the stoichiometric subspace. Hence the deficiency is indeed $\delta =N-1-(N-1)=0$.

### Long Time Behaviour

**Asymptotic stability**

By standard results on compartmental systems, since the truncated system (8) is strongly connected, there is a unique positive equilibrium (that is, a stationary PDF) ${\overline{p}}\in {\mathbb {R}}_+^N$ that attracts every admissible initial value (Maeda et al. 1978, Theorem 6).

#### Remark 2

As a conclusion of the above assertions a mass-action CRN can be assigned to the truncated conservative system (8) whose reaction graph is strongly connected and has deficiency zero. thus, the same assertion follows from CRN theory and, in particular, from the deficiency zero theorem (Feinberg 2019; Anderson 2011).

Furthermore, we also know that the system is Lyapunov stable with the standard entropy-like logarithmic Lyapunov function

$$\begin{aligned} V(p,\overline{p})=\sum _{\alpha }\left( p_{\alpha }\log \frac{p_{\alpha }}{\overline{p}_{\alpha }}+{\overline{p}}_{\alpha }-p_{\alpha }\right) . \end{aligned}$$

Finally, a well-known result (Maeda and Kodama 1979) shows that for two solutions *p*(*t*) and *q*(*t*) of (8), the following inequality holds:

$$\begin{aligned} \Vert {p(t_1)-q(t_1)} \Vert _{L^1}\le \Vert {p(t_2)-q(t_2)} \Vert _{L^1}\qquad t_1\ge t_2\ge 0. \end{aligned}$$

In particular, if we set $q={\overline{p}}$ this shows that the convergence to the unique equilibrium is monotone in the $L^1$ norm.

### Computing the Equilibrium

We can easily approximate ${\overline{p}}$ by simulating the system on an appropriately large time interval. However, such a simulation can be computationally expensive and it is not trivial to determine the necessary time interval. Furthermore, in many applications we may not be interested in the time evolution of the system, only in the equilibrium ${\overline{p}}$. Instead, relying on the linear nature of the system (8) we may explicitly compute the equilibrium with the following approach.

We can incorporate the conservation into the equilibrium condition as

9 $$\begin{aligned} {\hat{\Gamma }}^{(N)}{\overline{p}}=\begin{bmatrix}1\\ 0\\ 0\\ \vdots \\ 0\end{bmatrix}=:e_1 \end{aligned}$$

where ${\hat{\Gamma }}^{(N)}$ is obtained from $\tilde{\Gamma }^{(N)}$ by replacing the first row with $h{\textbf{1}}_N^{\textrm{T}}\in {\mathbb {R}}^N$. Since $\tilde{\Gamma }^{(N)}$ has a one-dimensional left kernel (by virtue of the rank-nullity theorem and the fact that zero is a simple eigenvalue, see Foster and Jacquez 1975) spanned by ${\textbf{1}}_N$, any $N-1$ rows are independent. To see this, assume by contradiction that not any $N-1$ rows are independent. Then there exists a nonzero vector in the left kernel of $\tilde{\Gamma }^{(N)}$ that has a zero coordinate, but then the left kernel cannot be spanned by ${\textbf{1}}_N$. Clearly ${\textbf{1}}_N$ is not in the left kernel of ${\hat{\Gamma }}^{(N)}$ and $\textrm{Im}\tilde{\Gamma }^{(N)}\subsetneq \textrm{Im}{\hat{\Gamma }}^{(n)}$, and thus $\textrm{rank}\,{\hat{\Gamma }}^{(N)}=N$, hence we can find the equilibrium ${\overline{p}}$ by simply solving the linear system of equations (9).

## Numerical Experiments

In this section we present some examples from the literature and discuss the memory requirement of the kinetic FVM.

### Examples

In this section we compare the performance of our method to that of SELANSI (Pájaro et al. 2017). For more information about the examples the reader is referred to Pájaro et al. (2017). The numerical simulations have been performed on a computer with Intel(R) Core(TM) i7-8565U CPU @ 1.80GHz and 16 GB of RAM in MATLAB R2022b. The solution (9) is solved with built-in iterative solvers. The final time and time step of the SELANSI simulations are noted for each example.

#### Example 1: Single Gene Self-Regulation with Positive Feedback

The first example is a GRN consisting of a single gene. The regulation is described by the Hill function

$$\begin{aligned} c_1(x_1)=\frac{K_1^{H_{11}}+\varepsilon _1x_1^{H_{11}}}{K_1^{H_{11}}+x_1^{H_{11}}}. \end{aligned}$$

We consider a negative Hill coefficient, corresponding to a positive self-regulation. In this case, as described in Pájaro et al. (2015) (see also Friedman et al. 2006) the stationary solution of (4) can be explicitly calculated as follows:

$$\begin{aligned} {\overline{p}}(x)=C\rho ^{\frac{k_m^1(1-\varepsilon _1)}{H_{11}}}(x)x^{-(1-k_m^1\varepsilon _1)}e^{-\frac{x}{b_1}}, \end{aligned}$$

where $\rho (x)=\frac{x^{H_{11}}}{K_1^{H_{11}}+x_1^{H_{11}}}$ and $C>0$ is a constant ensuring that ${\overline{p}}(x)$ integrates to one. As discussed before, the solution has a singularity at $x=0$, since it has a factor of $x^{-(1-k_m^1\varepsilon _1)}$. Clearly the exponent is larger than $-1$, hence the average of the solution over the first mesh cell will be finite.

**Fig. 2:**
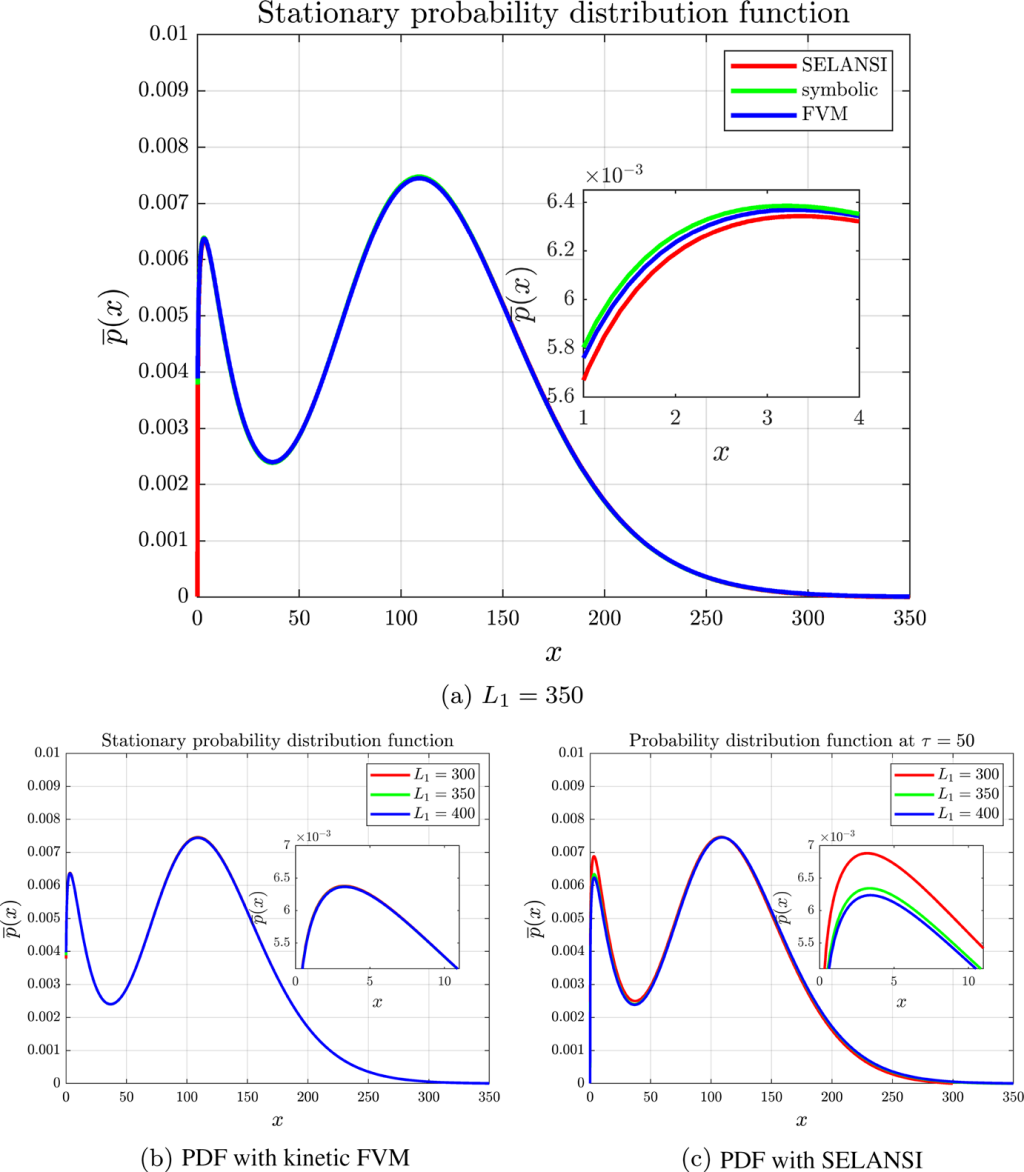
Self-regulated single gene network with parameters $H_{11}=-4$, $K_1=45$, $\varepsilon _1=0.15$, $k_m^1=3.2\times 10^{-3}$, $b_1=16$ and $\gamma _x^1(x)=4\times 10^{-4}$. The simulations are performed with $N_1=2000$, $\tau =t\gamma _x^1=50$ and $\Delta t=0.002$ (color figure online)

Figure 2 shows the simulation results for various $L_1$ values. Observe that if the domain size is set ideally (Fig. 2a), then both approaches provide a good approximation of the symbolic solution. However, SELANSI cannot handle cases where the domain is too small or too large and skews the solution, see the highlighted section of Fig. 2c. This is assumed to be because (i) SELANSI imposes zero Dirichlet boundary conditions, (ii) the semilagrangian method is not conservative, thus SELANSI has to manually renormalize in each iteration. Compared to this, our kinetic FVM is nonnegative and conservative and, as noted in Sect. 4, the equilibrium is strictly positive. Thus it can capture the qualitative behaviour of the PIDE even if the domain is not known precisely, which might be the case for previously untested gene regulatory network structures or parameter sets, see Fig. 2b. Table 1 shows the relative error (in the $L^2$ norm) of the different methods compared to the symbolic solution, computed as follows:

$$\begin{aligned} E(p,p_{ref})=\frac{\Vert {p-p_{ref}} \Vert _{L^2}}{\Vert {p_{ref}} \Vert _{L^2}}=\frac{\sqrt{\sum _{i=1} ^N\left( p(x_i)-p_{ref}(x_i)\right) ^2}}{\sqrt{\sum _{i=1}^Np_{ref}^2(x_i)}}. \end{aligned}$$

The computational times of both methods are depicted in Table 2, from where we can observe that the FVM has also better computational efficiency compared to that of SELANSI.

#### Example 2: Mutual Activation of Two Genes

**Fig. 3:**
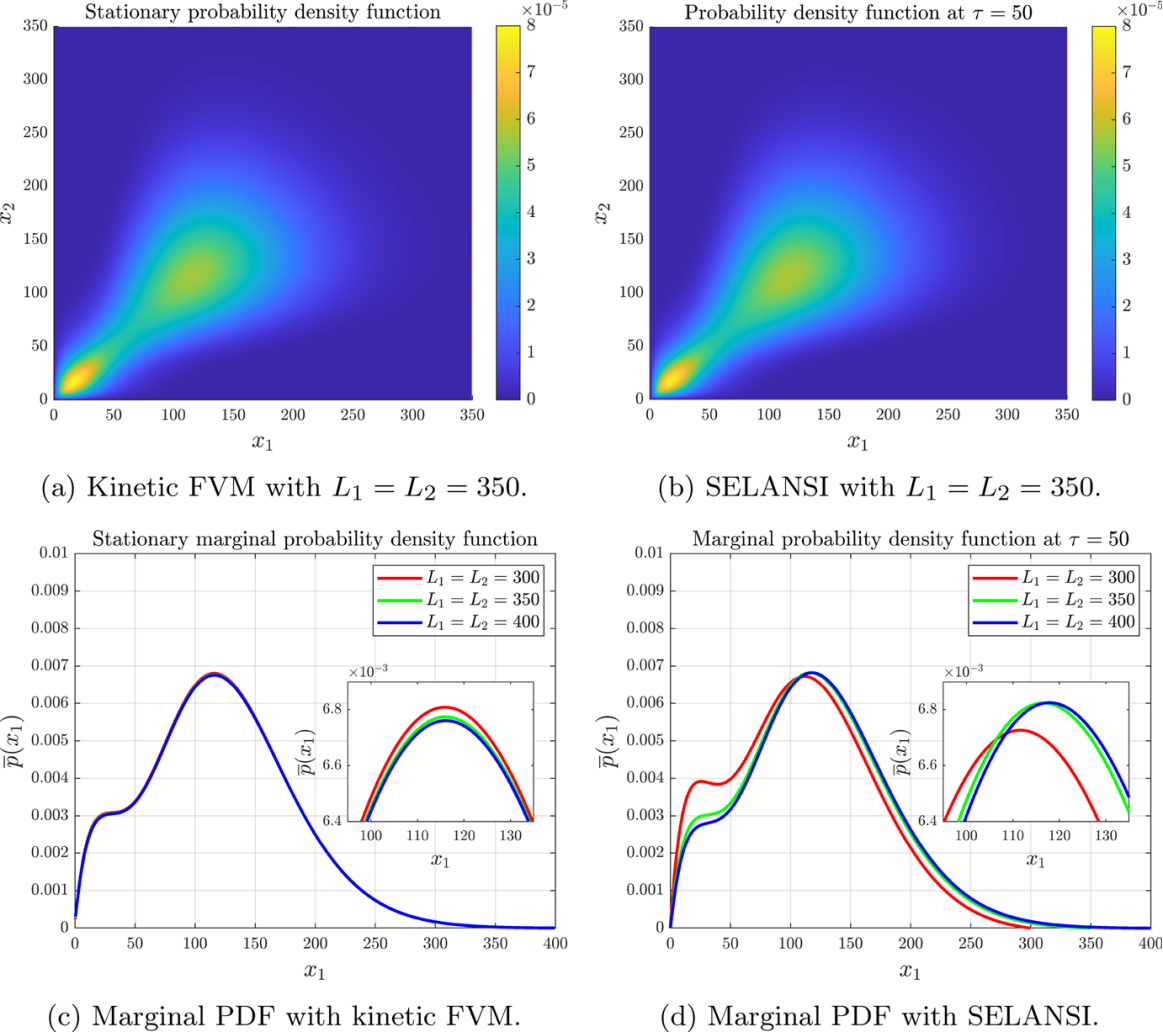
Mutual activation with parameters $H_{12}=H_{21}=-4$, $K_{12}=K_{21}=70$, $\varepsilon _1=\varepsilon _2=0.2$, $k_m^1=k_m^2=3.4\times 10^{-3}$, $b_1=b_2=18$, $\gamma _x^1(x)=\gamma _x^2(x)=4\times 10^{-4}$, $N_1=N_2=400$, $\tau =t\gamma _x^1$=50 and $\Delta t=0.005$ (color figure online)

In this example we consider Hill functions in the form of

$$\begin{aligned} \begin{aligned} c_1({\varvec{x}})&=\frac{K_{12}^{H_{12}}+\varepsilon _1x_2^{H_{12}}}{K_{12}^{H_{12}}+x_2^{H_{12}}},\\ c_2({\varvec{x}})&=\frac{K_{21}^{H_{21}}+\varepsilon _2x_1^{H_{21}}}{K_{21}^{H_{21}}+x_1^{H_{21}}}, \end{aligned} \end{aligned}$$

where $H_{12}<0$ and $H_{21}<0$, corresponding to positive cross-regulation or activation. Figure 3a, b respectively show the stationary joint PDF, while Fig. 3c, d respectively show the stationary marginal PDF on various domains, for FVM and SELANSI. Note, that the GRN is symmetric w.r.t. the proteins, thus we only plot one set of marginal PDFs. We can observe the sensitivity of SELANSI to the domain, while the finite volume discretization is quite robust to it. In this example we can see that the solution computed by SELANSI deteriorates not just for too small, but even for too large domains. Since for multidimensional GRNs the symbolic solution of (1) cannot be computed in a straightforward manner, we cannot compute the empirical error as in the case of the one-dimensional example. Instead, we only compare the running times of the two methods, the results of which are collected in Table 3.

**Table 1:** Relative error of the simulation of a one-dimensional GRN on various domains

Mesh	FVM ($\times 10^{-3}$)			SELANSI ($\times 10^{-3}$)		
	$L_1=300$	$L_1=350$	$L_1=400$	$L_1=300$	$L_1=350$	$L_1=400$
$2.5\times 10^4\times 800$	7.4358	8.6799	9.9106	29.6010	5.4881	9.2314
$2.5\times 10^4\times 1200$	4.9886	5.8169	6.6366	28.6252	4.3113	8.3388
$2.5\times 10^4\times 1600$	3.7665	4.3870	5.0013	29.5308	3.4255	8.0746
$2.5\times 10^4\times 2000$	3.0335	3.5298	4.0209	29.9408	2.8899	7.2511
$2.5\times 10^4\times 5000$	1.4271	1.4761	1.6675	31.0859	2.5960	6.0042

**Table 2:** Average runtime of 100 simulations of a one-dimensional GRN with various mesh sizes

Mesh	FVM	SELANSI
$2.5\times 10^4\times 800$	0.0335 *s*	1.8208 *s*
$2.5\times 10^4\times 1200$	0.0899 *s*	2.1993 *s*
$2.5\times 10^4\times 1600$	0.1460 *s*	2.5510 *s*
$2.5\times 10^4\times 2000$	0.3561 *s*	2.8866 *s*
$2.5\times 10^4\times 5000$	3.8066 *s*	7.9131 *s*

#### Example 3: Mutual Repression of Two Genes

**Fig. 4:**
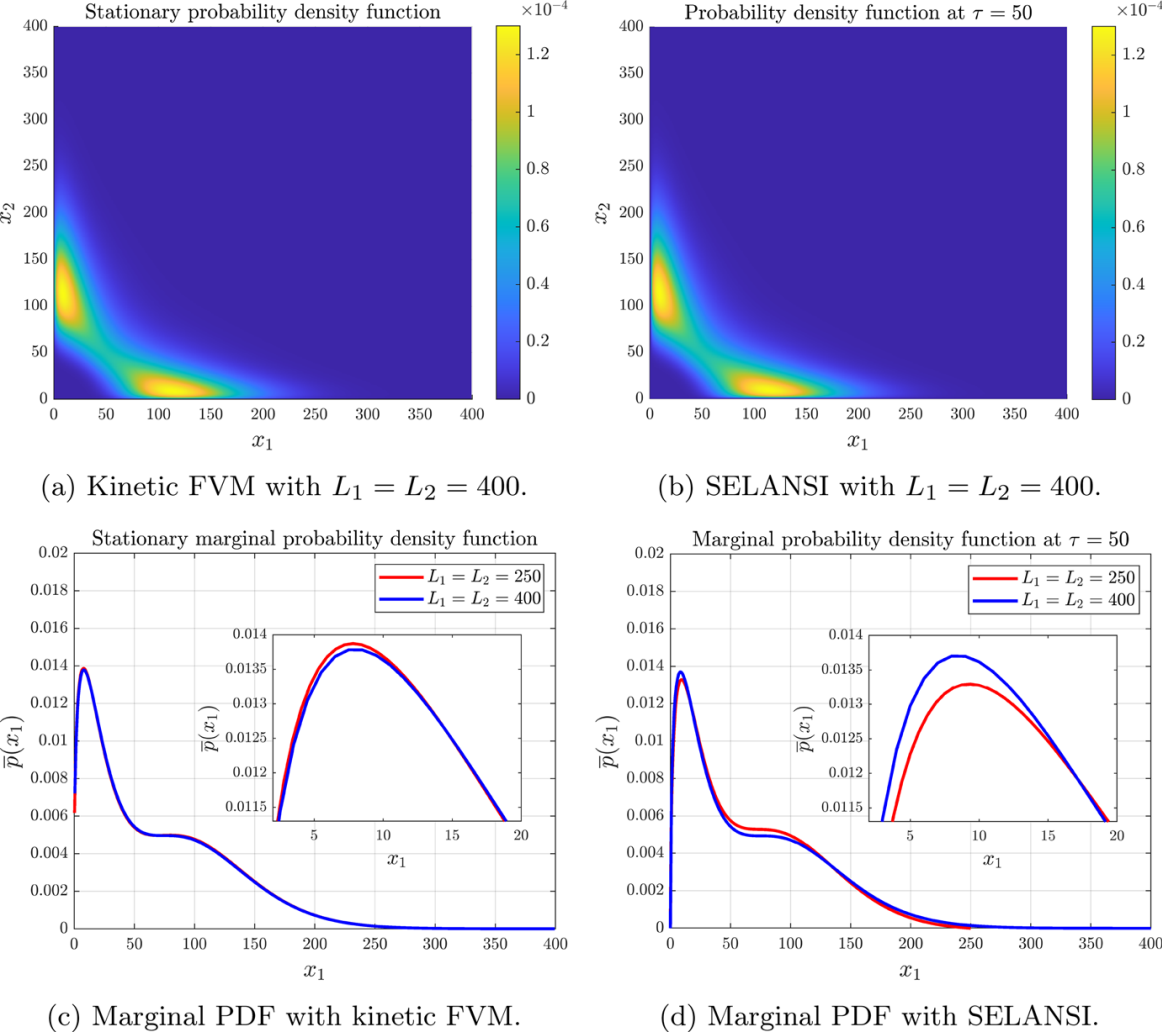
Mutual repression with parameters $H_{12}=H_{21}=4$, $K_{12}=K_{21}=45$, $\varepsilon _1=\varepsilon _2=0.15$, $k_m^1=k_m^2=3.2\times 10^{-3}$, $b_1=b_2=16$, $\gamma _x^1(x)=\gamma _x^2(x)=4\times 10^{-4}$, $N_1=N_2=400$, $\tau =t\gamma _x^1=50$ and $\Delta t=0.005$ (color figure online)

In this example we consider Hill functions in the form of

$$\begin{aligned} c_1({\varvec{x}})=\frac{K_{12}^{H_{12}}+\varepsilon _1x_2^{H_{12}}}{K_{12}^{H_{12}}+x_2^{H_{12}}},\qquad c_2({\varvec{x}})=\frac{K_{21}^{H_{21}}+\varepsilon _2x_1^{H_{21}}}{K_{21}^{H_{21}}+x_1^{H_{21}}}, \end{aligned}$$

where $H_{12}>0$ and $H_{21}>0$, corresponding to negative cross-regulation or repression. Figure 4 shows the stationary joint PDF and the marginal stationary PDF on multiple domains. Again, the GRN is symmetric w.r.t. the proteins and the same dependence on the domain can be observed in the case of SELANSI. The running time of both methods with various mesh sizes are presented in Table 3.

#### Example 4: Self and Mutual Regulation

**Fig. 5:**
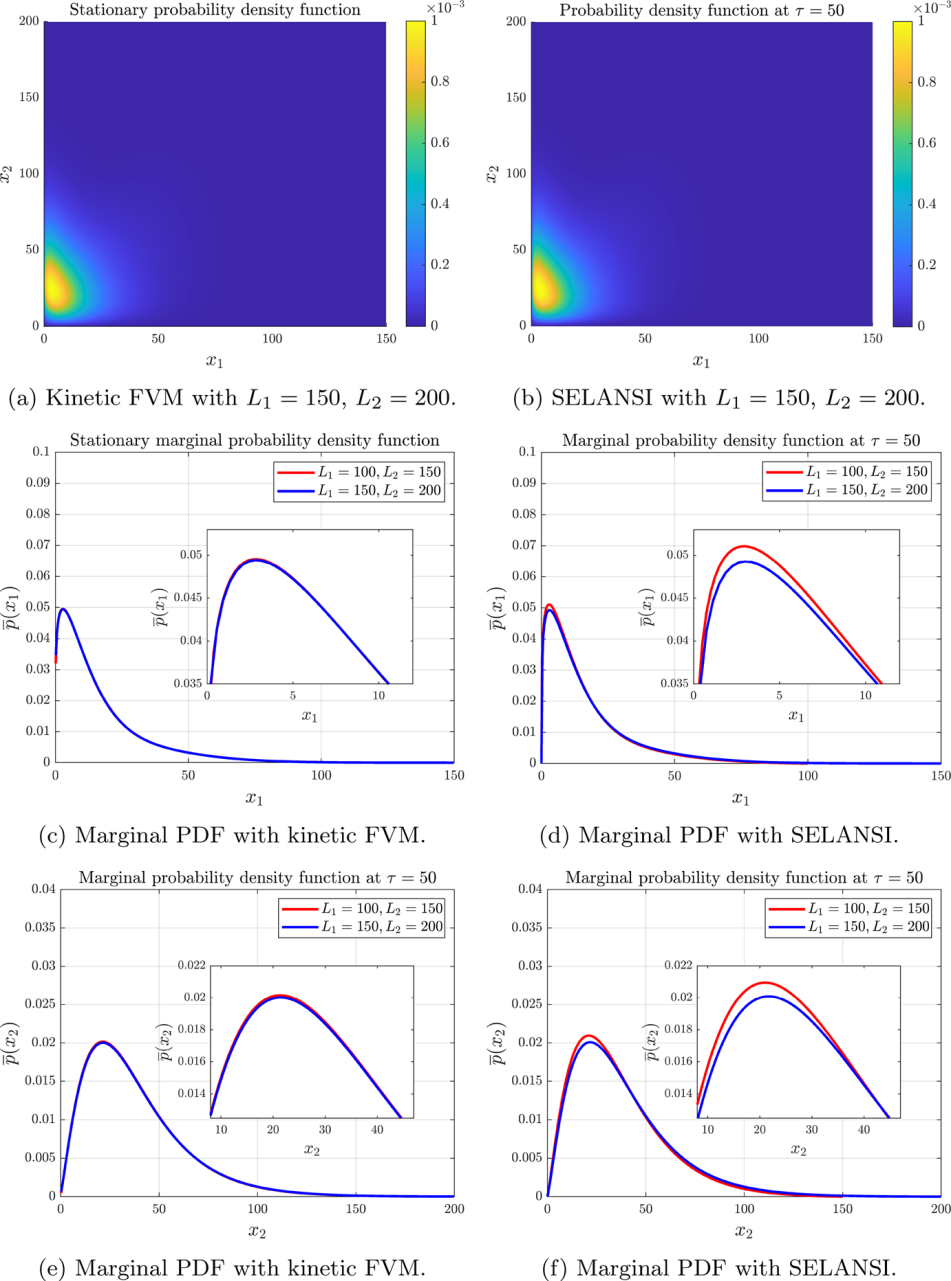
Mutual repression with parameters $H_{11}=-4$, $H_{21}=-6$, $H_{12}=H_{22}=2$, $K_{11}=K_{12}=45$, $K_{21}=K_{22}=70$, $\varepsilon _{11}=\varepsilon _{21}=0.002$, $\varepsilon _{12}=0.02$, $\varepsilon _{22}=0.1$, $\varepsilon _{13}=\varepsilon _{23}=0.2$, $k_m^1=4\times 10^{-3}$, $k_m^2=8\times 10^{-3}$, $b_1=10$, $b_2=20$, $\gamma _x^1(x)=\gamma _x^2(x)=4\times 10^{-4}$, $N_1=N_2=400$, $\tau =t\gamma _x^1=50$ (color figure online)

In this example we consider two genes, one of which is activated by both, the other is repressed by both. The corresponding Hill functions can be given as follows:

$$\begin{aligned} \begin{aligned} c_1({\varvec{x}})&=\frac{\varepsilon _{11}x_1^{H_{11}}x_2^{H_{12}} +\varepsilon _{12}K_{11}^{H_{11}}x_2^{H_{12}}+\varepsilon _{13}x_1^{H_{11}}K_{12}^{H_{12}} +K_{11}^{H_{11}}K_{12}^{H_{12}}}{x_1^{H_{11}}x_2^{H_{12}}+K_{11}^{H_{11}}x_2^{H_{12}} +x_1^{H_{11}}K_{12}^{H_{12}}+K_{11}^{H_{11}}K_{12}^{H_{12}}},\\ c_2({\varvec{x}})&=\frac{\varepsilon _{21}x_1^{H_{21}}x_2^{H_{22}} +\varepsilon _{23}K_{21}^{H_{21}}x_2^{H_{22}}+\varepsilon _{12}x_2^{H_{21}}K_{22}^{H_{22}} +K_{21}^{H_{21}}K_{22}^{H_{22}}}{x_1^{H_{21}}x_2^{H_{22}}+K_{21}^{H_{21}}x_2^{H_{22}} +x_2^{H_{21}}K_{22}^{H_{22}}+K_{21}^{H_{21}}K_{22}^{H_{22}}}, \end{aligned} \end{aligned}$$

where $H_{11}<0$, $H_{21}<0$, $H_{12}>0$ and $H_{22}>0$. We note that the above functions are generalized Hill functions, and thus have to be defined in a separate file for the SELANSI simulation. Figure 5 shows the stationary joint PDF and the marginal stationary PDF on multiple domains. This example is not symmetric w.r.t. the different kind of proteins, thus we plot both marginal density functions. The running time of both methods with various mesh sizes are presented in Table 3.

**Table 3:** Average runtime of 100 simulations of several two-dimensional GRNs with various mesh sizes

GRN	FVM				SELANSI			
	$100^2$	$200^2$	$300^2$	$400^2$	$100^2$	$200^2$	$300^2$	$400^2$
Ex. 2	0.2223 *s*	1.6485 *s*	5.2159 *s*	11.5707 *s*	9.0423 *s*	21.5449 *s*	40.6726 *s*	71.1792 *s*
Ex. 3	0.2128 *s*	1.3679 *s*	4.5709 *s*	10.3817 *s*	9.0443 *s*	21.3982 *s*	40.7456 *s*	72.3397 *s*
Ex. 4	0.3241 *s*	1.8639 *s*	5.7925 *s*	12.3953 *s*	9.1355 *s*	22.0259 *s*	41.9351 *s*	73.4001 *s*
Ex. 5	0.7064 *s*	4.9921 *s*	17.0973 *s*	45.0809 *s*	−	−	−	−

#### Example 5: Bacterial Competence

In *Bacillus subtilis*, competence is a probabilistic and transiently differentiated state. In this physiological state bacteria has the capability of DNA uptake from their environment. The phenomena is modelled with a two-dimensional gene regulatory network, consisting of the master regulator self-activated ComK which represses the transcription factor ComS (Süel et al. 2007). Protein degradation is mediated by the MecA complex. After ComK (ComS) binds to the complex an intermediate complex MecA-ComK (MecA-ComS) complex is formed, in which the ComK (ComS) protein is degraded by the ClpP-ClpC proteases (Süel et al. 2006). Instead of explicitly modelling the effects of the MecA complex, the authors consider a variable degradation rate. Using this reduced order stochastic differential equation developed in Süel et al. (2006) a discrete stochastic CME model is presented in Dandach and Khammash (2010), simulated using a Monte-Carlo based Stochastic Simulation Algorithm. A corresponding PIDE is presented in Pájaro et al. (2017) with parameters adapted from the CME model of Dandach and Khammash (2010) as follows: $\alpha _k=0.0028$, $\beta _k=0.049$, $\beta _s=0.057$, $K_k=100$, $K_s=110$, $\delta _k=\delta _s=0.0014$, $\Gamma _k=500$, $\Gamma _s=50$, $b_1=2$, $b_2=5$, $k_m^1=\frac{\alpha _k+\beta _k}{b_1}$, $k_m^2=\frac{\beta _s}{b_2}$, $\varepsilon _1=\frac{\alpha _k}{\alpha _k+\beta _k}$, $\varepsilon _2=0$, $H_{11}=-2$, $H_{21}=5$. The coefficient functions are set as:

$$\begin{aligned} \begin{aligned} c_1({\varvec{x}})&=\frac{K_k^{H_{11}}+\varepsilon _1x_1^{H_{11}}}{K_k^{H_{11}} +x_1^{H_{11}}},\qquad \gamma _x^1({\varvec{x}})=\frac{\delta _k\Gamma _k\Gamma _s}{\Gamma _k\Gamma _s+\Gamma _sx_1+\Gamma _kx_2},\\ c_2({\varvec{x}})&=\frac{K_s^{H_{21}}+\varepsilon _2x_1^{H_{21}}}{K_s^{H_{21}} +x_1^{H_{21}}},\qquad \gamma _x^2({\varvec{x}})=\frac{\delta _s\Gamma _k\Gamma _s}{\Gamma _k\Gamma _s+\Gamma _sx_1+\Gamma _kx_2}. \end{aligned} \end{aligned}$$

**Fig. 6:**
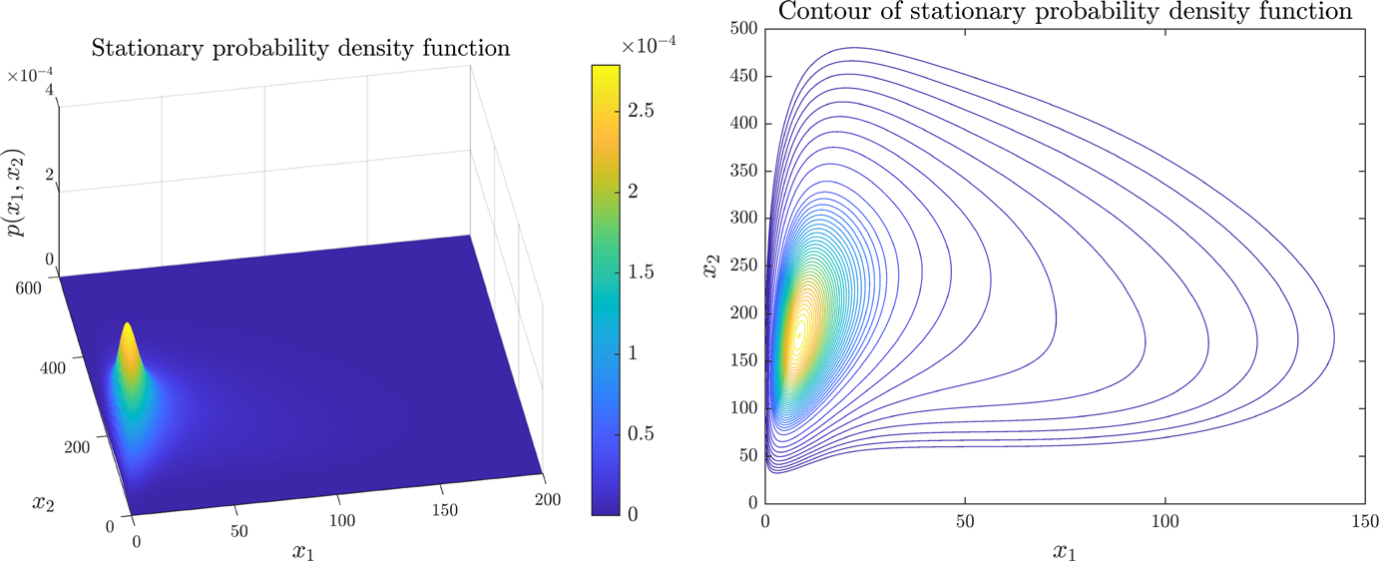
Kinetic FVM of Example 5 with $N_1=N_2=400$ (color figure online)

We note that the currently publicly available SELANSI version cannot handle variable degradation rates, thus we could not reproduce the plots of Pájaro et al. (2017). Figure 6 shows the stationary PDFs and its contour plot, both of which are in accordance with the plots of Pájaro et al. (2017). The running times of the kinetic FVM for various mesh sizes are shown in Table 3.

### Memory Requirement of the Kinetic FVM

A notable technical challenge in our method is the efficient assembly and storage of the coefficient matrix $\Gamma ^{(N)}$. For $n\ge 2$ one should store $\Gamma ^{(N)}$ in a sparse representation, but even then the memory requirement can grow quickly. However, we can explicitly calculate the number of nonzero elements of the matrix to aid the design of the simulation. To be precise, the number of nonzero elements of the coefficient matrix corresponding to an *n*-dimensional PIDE discretized on a grid of size $\prod _{i=1}^nN_i$ is as follows:

$$\begin{aligned}&\sum _{i=1}^n\underbrace{\left( \sum _{k=1}^{N_i}(N_i-k)\right) \prod _{\begin{array}{c} j=1\\ j\ne i \end{array}}^nN_j}_{\text {bursting of protein }X_i}+\sum _{i=1}^n\underbrace{(N_i-1)\prod _{\begin{array}{c} j=1\\ j\ne i \end{array}}^nN_j}_{\text {degradation of }X_i}+\underbrace{\prod _{i=1}^nN_i}_{\text {diagonal}}\\&\quad =\sum _{i=1}^n\frac{1}{2}\left( N_i^2-N_i\right) \prod _{\begin{array}{c} j=1\\ j\ne i \end{array}}^nN_j+n\prod _{i=1}^nN_i-\sum _{i=1}^n\prod _{\begin{array}{c} j=1\\ j\ne i \end{array}}^nN_j+\prod _{i=1}^nN_i\\&\quad =-\frac{1}{2}n\prod _{i=1}^nN_i+\frac{1}{2}\left( \sum _{i=1}^nN_i\right) \left( \prod _{i=1}^nN_i\right) +(n+1)\prod _{i=1}^nN_i-\left( \sum _{i=1}^n\frac{1}{N_i}\right) \left( \prod _{i=1}^nN_i\right) \\&\quad =\frac{1}{2}\left( n+2+\sum _{i=1}^nN_i-\sum _{i=1}^n\frac{2}{N_i}\right) \left( \prod _{i=1}^nN_i\right) . \end{aligned}$$

**Fig. 7:**
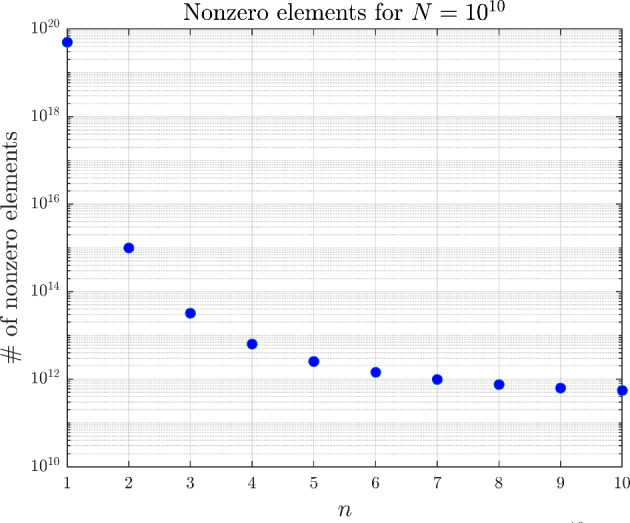
Memory requirement of an *n*-dimensional GRN with $N=10^{10}$ total number of cells

Figure 7 shows the number of nonzero elements on an equidistant grid for a matrix corresponding to a mesh of size $N=10^{10}$ (that is, the matrix has $10^{20}$ total elements) as a function of *n*. The logarithmic scaling suggests that as the dimension of the PIDE is increased, we can increase the number of finite volume cells on the grid even without exceeding the memory limits.

## Conclusions

A novel discretization scheme was proposed in this paper for the simulation and analysis of multidimensional PIDE models used in the stochastic dynamical description of gene regulatory networks. It was shown that using an appropriate finite volume scheme, a fully conservative linear compartmental dynamics is obtained in ODE form. This representation can be particularly useful for studying the dynamics of the process from a systems biology perspective, since the interpretation of the system of ODEs of a process is usually more intuitive than the original PIDE. In particular, the proposed compartmental description can serve as a basis for designing experiments and solving various analysis and control problems of stochastic gene regulatory networks, since the theoretical properties of the model class are well-known regardless of the dimension of the state variable (van Kampen 2007). The interconnection structure of the discretized system was studied in detail, and it was shown that the associated directed graph is always strongly connected. Therefore, the theory of kinetic and compartmental systems can be used to conclude that the equilibrium of the discretized dynamics representing the stationary distribution of molecules is unique and globally stable for any biologically meaningful parameter values in the PIDE model. Moreover, the stationary distribution can be obtained by solving a set of linear equations without performing the time-domain simulation. The memory requirement of the method can be precisely pre-computed based on which the applicable resolution can be determined. Five illustrative examples were presented to show the operation and performance of the method. Whenever possible, the obtained solutions and running times were compared with those given by the SELANSI toolbox, and these comparisons clearly justified the advantageous properties of the proposed approach both in terms of precision and performance. Further work will be focused on feedback control design based on the semidiscretized models.

## Data Availability

All data generated or analysed during this study are included in this published article. Our implementation along with the examples found in this manuscript can be found at the following GitHub repository: mihalyvaghy/PIDE.
